# Role of Acoustic Streaming in Formation of Unsteady Flow in Billet Sump during Ultrasonic DC Casting of Aluminum Alloys

**DOI:** 10.3390/ma12213532

**Published:** 2019-10-28

**Authors:** Sergey Komarov, Takuya Yamamoto

**Affiliations:** Graduate School of Environmental Studies, Tohoku University, Miyagi 980-8579, Japan; takuya.yamamoto.e6@tohoku.ac.jp

**Keywords:** ultrasonic DC casting, acoustic streaming, aluminum alloy, mathematical model, unsteady flow phenomena, sump evolution, mushy zone, experimental verification

## Abstract

The present work investigated melt flow pattern and temperature distribution in the sump of aluminum billets produced in a hot-top equipped direct chilling (DC) caster conventionally and with ultrasonic irradiation. The main emphasis was placed on clarifying the effects of acoustic streaming and hot-top unit type. Acoustic streaming characteristics were investigated first by using the earlier developed numerical model and water model experiments. Then, the acoustic streaming model was applied to develop a numerical code capable of simulating unsteady flow phenomena in the sump during the DC casting process. The results revealed that the introduction of ultrasonic vibrations into the melt in the hot-top unit had little or no effect on the temperature distribution and sump profile, but had a considerable effect on the melt flow pattern in the sump. Our results showed that ultrasound irradiation makes the flow velocity faster and produces a lot of relatively small eddies in the sump bulk and near the mushy zone. The latter causes frequently repeated thinning of the mushy zone layer. The numerical predictions were verified against measurements performed on a pilot DC caster producing 203 mm billets of Al-17%Si alloy. The verification revealed approximately the same sump depth and shape as those in the numerical simulations, and confirms the frequent and large fluctuations of the melt temperature during ultrasound irradiation. However, the measured temperature distribution in the sump significantly differed from that predicted numerically. This suggests that the present mathematical model should be further improved, particularly in terms of more accurate descriptions of boundary conditions and mushy zone characteristics.

## 1. Introduction

Ultrasonic casting is receiving increasingly more attention, because it provides relatively simple and effective way to control solidification structure of various alloys. There has been an especially great progress in the development of ultrasonic casting technology for light metals, particularly aluminum alloys. The main achievements in this area have has been summarized in a number of books [1,2] and review papers [3,4]. It is well known from the relevant literature that most of the ultrasound-assisted technologies exploit acoustic cavitation, the phenomenon of nucleation, growth, and violent implosion of tiny bubbles. There is a large volume of literature devoted to acoustic cavitation, especially in fields such as sonochemistry, wastewater treatment, biotechnology, and mineral engineering [4,5,6,7,8].

Stable cavitation starts when the sound pressure exceeds a threshold value. This value is dependent on such factors as type of liquid, content of gas, and concentration of solid impurities in liquid. For instance, in tap water and commercially pure molten aluminum, this threshold was found to be attained when the peak-to-peak amplitudes of sonotrode vibrations becomes higher than 4–5 μm and 10–11 μm, respectively, at a frequency of 20 kHz [9]. Generally, such a low vibration amplitude can be produced using many types of commercially available ultrasonic equipment. However, when the treatment process needs to be scaled up to meet industrial requirements, a number of serious challenges arise. The first, and perhaps most difficult challenge, is that the size and shape of sonotrode, which is one of the main parts of powerful ultrasonic installations, cannot be arbitrarily changed. It is commonly known that acoustic cavitation occurs over a limited space near the ultrasonic radiating surface. However, a simple enlargement of the sonotrode area presents a considerable challenge, because the sonotrode design must meet resonance conditions imposing strict constraints on its size and shape. These constraints are especially severe for ultrasonic-assisted casting, because in this case the sonotrode material must resist well high-temperature cavitation erosion, thermal shock, and high temperature oxidation.

Another problem is occurrence of acoustic streaming, which is inevitably generated when ultrasound is irradiated in a liquid, especially at a commonly used frequency of 20 kHz. Acoustic streaming is defined as a steady fluid motion caused by the attenuation of ultrasonic waves in fluids. Although there are three types of acoustic streaming depending on the streaming scale, this study focused only on the large-scale streaming, because this type of streaming has the greatest effect on the liquid flow near the sonotrode. The main problem resulting from the acoustic streaming is that liquid flow near the sonotrode becomes hardly controllable. Particularly, in continuous or semi-continuous ultrasonic casting, i.e., when molten metal can pass through the cavitation zone only once, the flow control near the sonotrode surface is one of the key factors influencing the process efficiency. A typical example is the direct chilling (DC) casting process in the aluminum industry. In this case, the best flow condition would be to ensure that all or at least most of the amount of the molten metal can be passed through the zone of intense cavitation located near the sonotrode working tip. This condition is especially important when ultrasonic vibrations are applied to the melt aiming at dispersing particles of grain refiners.

Another feature related to the large-scale acoustic streaming may be a forced convection of liquid metal which may affect the heat transfer during the metal solidification process. This may cause problems when ultrasound is irradiated into a pool of solidifying melt, for example in the DC casting process. In this case, the heat convection may result in a temperature drop in the molten metal causing a deterioration of its solidification structure. This problem has been briefly discussed in our earlier investigation on the ultrasonic DC casting of Al-Si hypereutectic alloys [10]. These two issues, namely the effects of acoustic streaming on the passage of melt through the cavitation zone and on heat convection, suggest that the acoustic streaming should be properly controlled in ultrasonic-assisted casting processes. However, our understanding of the underlying relationships influencing the above phenomena still remains unclear.

It should be noted that a lot of results on acoustic streaming and its related phenomena have been published recently in sonochemical literature. Although water or aqueous solutions were used as a liquid in these studies, it is a reasonable assumption that the features of acoustic streaming and mechanisms of its generation in water and molten aluminum are very similar. At least, the similarity of cavitation in water and aluminum was suggested in the earlier study [11]. Some features and underlying mechanisms of acoustic streaming generation have been investigated also in our earlier paper [12] both experimentally and numerically. In this paper, based on the well documented fact that acoustic cavitation and acoustic streaming are interrelated phenomena, a mathematical model of acoustic streaming has been proposed and experimentally verified by using water model and PIV (Particle Image Velocimetry) technique. Moreover, this paper contains a brief review of recent results of the above mentioned studies dealing with the sonochemical technology. Later on, the proposed model was used to numerically predict the size of cavitation zone and velocity of acoustic streaming in molten aluminum [13]. The results reveal that due to large difference in physical and acoustic properties, in aluminum melt attenuation of sound waves is larger and velocity of acoustic streaming is smaller than those in water if the sound pressure amplitude at the sonotrode tip is the same.

Nevertheless, studies on the acoustic streaming in molten aluminum and its effects on the solidification and structure of aluminum alloys are still scarce. The mere fact that acoustic streaming affects the solidification structure of aluminum alloys has long been known. Results of recent and earlier studies concerning the acoustic streaming in molten metals have been summarized in the above cited books [1,2]. However, experimental difficulties in measuring flow velocity under very high temperatures have limited earlier attempts to investigate the acoustic streaming in molten metals. Although experimental investigations of acoustic streaming in molten metals still predict a big challenge, current progress in supercomputer’s capabilities and CFD techniques has made possible very accurate and realistic numerical simulation of multiphase fluid flows, heat, and mass transfer phenomena including the acoustic flows in bubbly liquids and molten metals as well as solidification phenomena. Thus, numerical simulation in combination with cold physical modeling, for example using water models, provides a very powerful tool for investigating the ultrasonic-assisted casting processes.

As mentioned above, acoustic streaming in molten metals and its underlying mechanisms have much in common with those in other liquids including water. Therefore, the results of earlier studies [14,15,16] on numerical simulation of acoustic streaming in water systems are of considerable importance for ultrasonic casting technology. Additionally, the recent works of Eskin et al. [17,18,19] have made a great contribution in understanding the mechanism and features of acoustic streaming in molten aluminum. For instance, in one of the studies [19], they developed a mathematical model to simulate an acoustic flow in the sump of a DC caster, and on this basis predicted that the acoustic flow causes an increase in the cooling rate at the solidification front, which eventually results in refinement of solidification structure of A6060 alloy. It is noteworthy that this significant effect of microstructure refinement was achieved despite a relatively small and low-amplitude sonotrode used in this study.

The goal of the present study is to investigate the effects of acoustic streaming on the distribution of temperature and flow pattern in the sump of DC cast billets when different types of hot-top unit are used. As we have reported in our previous study [10], the effects of ultrasonic treatment of molten aluminum are very dependent on what fraction of melts can be passed through the cavitation zone, which is the more intensive in the immediate vicinity of sonotrode tip. This is especially important when the ultrasound vibrations are applied to disperse and activate refiner particles immediately before solidification or during the casting of metal. The present study includes three parts. In the first part, acoustic streaming was investigated experimentally using water models. The second part was devoted to development of mathematical model to simulate ultrasonic-assisted DC casting with emphasis on flow and heat transfer in the sump. In the third part, the model proposed was verified by using the results obtained in a pilot-scale DC caster.

## 2. Experimental Methods and Procedure 

### 2.1. Ultrasonic Equipment

The ultrasonic system used in this study comprises a commercially available ultrasonic generator (DG2000, Telsonic, Zurich, Switzerland) equipped with a piezoceramic converter and a dumbbell-shaped sonotrode made of Si_3_N_4_-based ceramics. Details on the sonotrode specification and characteristics can be found in our previous paper [20]. The working frequency of the ultrasonic system was automatically tuned to a frequency in the range of 18.9–21.2 kHz. The vibration amplitude of sonotrode tip was increased from 25 to 70 μm (p-p) in proportion to increasing the generator electric output power from 50 to 100%. The maximum power of generator was 2 kW. The amplitude was measured in air by using a Laser Displacement Sensor (LK-G5000, Keyence, Osaka, Japan).

### 2.2. PIV Measurements

**Figure 1:**
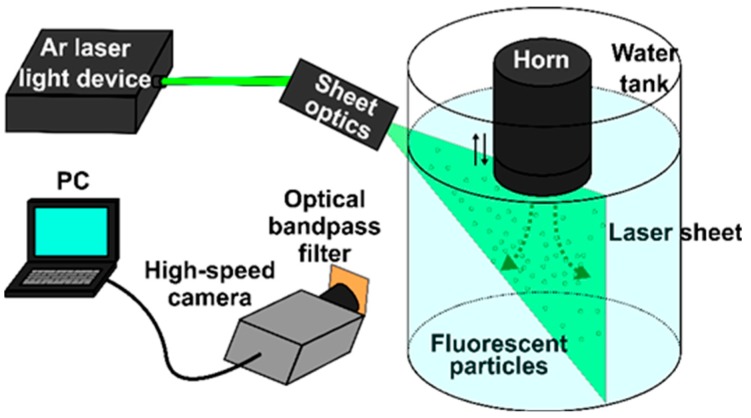
Experimental setup for Particle Image Velocimetry (PIV) measurements.

**Figure 2:**
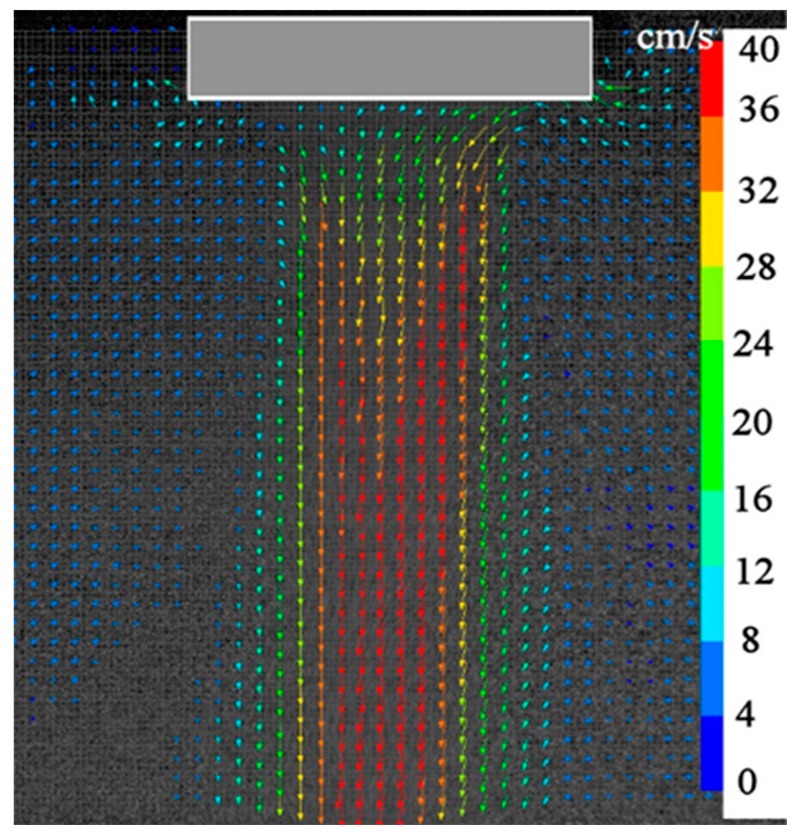
Typical results of PIV measurements.

Particle Image Velocimetry (PIV) system was applied to measure the acoustic streaming velocity. Figure 1 is a schematic drawing of the experimental setup. A rectangular glass vessel (W 30 × L 30 × D 40 cm^3^) was filled with water. Fluorescent particles (FLUOSTAR 0459, Kanomax Japan Inc., Shimizu, Japan) in an amount of 0.1 g were added to the water bath as a tracer. The average diameter of particles was 15 μm, and density was very close to that of water. The sonotrode tip was immersed vertically into the water bath to introduce ultrasonic vibrations followed by generation of cavitation and acoustic streaming. A water-cooled Ar lighting source (Optronics, Co., Ltd, Tokyo, Japan) was exploited to produce a visual laser sheet through the water bath in the immediate vicinity of the sonotrode tip, as shown in Figure 1. A high-speed camera (Photron, Tokyo, Japan) was used to record the tracer particle movement at a frame rate of 500 fps and a shutter time of 1/1000 second. When the fluorescent particles are illuminated by the Ar green light laser (wavelength 550 nm), they emit bright and well discernible light at a wavelength of 580 nm. This technique allowed us to completely eliminate any influence of cavitation bubbles on the PIV measurements. Then, the images taken were processed by a flow analysis software (Library Co., Ltd, Tokyo, Japan) to evaluate the acoustic streaming vector field. Figure 2 show typical results on the distribution of acoustic streaming velocity measured at a vibration amplitude of 50 μm (p-p). A comparison of these results with these published in our earlier paper [12] reveals that the use of fluorescent particles makes it possible to obtain much clearer picture of acoustic streaming vectors even in the immediate vicinity of the sonotrode tip.

### 2.3. Pilot DC Caster Tests

**Figure 3:**
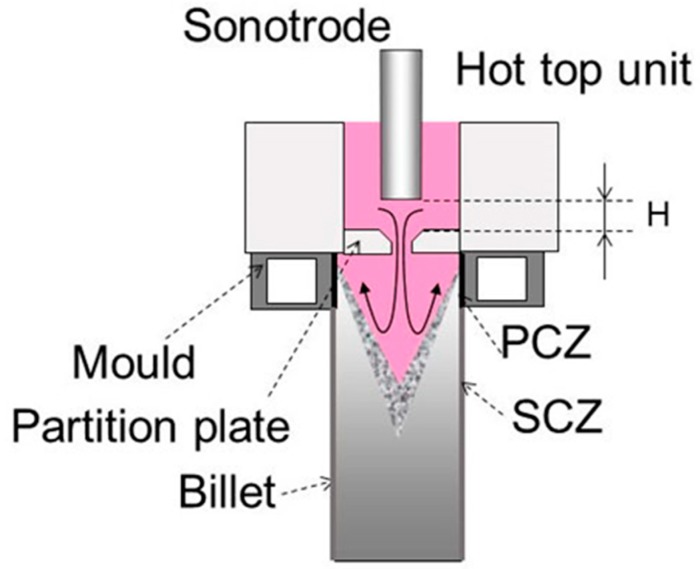
A schematic representation of flow-restricting hot-top for ultrasonic treatment.

Pilot vertical DC casting facility was utilized in the present investigation using an Al-17%Si model alloy. The alloy was melted in an electrical resistance furnace and commonly used Al-Cu-P refiner was added to the melt. After degassing with a rotary argon degasser unit, the melt was poured through a launder into the hot-top equipped DC caster mold to produce billets of 203 mm in diameter and about 2 m in length by using conventional or ultrasonic-assisted casting techniques. In the latter case, the following two methods of ultrasonic treatment (hereinafter referred as to “UST”) were examined. 1–UST inside hot-top, (UST HT) 2–UST inside the hot-top equipped by a bottom plate with a hole drilled at the plate center to let the melt flow in the mold at the center part of billet. This casting method will be referred as to UST in “flow-restricting hot-top” or UST FRHT. The DC caster of the latter type is schematically depicted in Figure 3. The sonotrode tip was preheated up to the melt temperature before its immersion into the melt. More details about these casting methods and the hot-top design can be found in our earlier study [10].

In order to verify the results of numerical simulation, the profile of sump and distribution of the melt temperature in sump were measured in a number of experiments. In the profile measurements, a certain amount of Al-30%Cu master alloy was premelted in a separate furnace and hold at the temperature of Al-Si melt used for casting. In the final stage of the casting, the Al-Si melt supply was stopped and replaced with the Al-Cu melt flowing into the caster mold over a short period of time. Then, the produced billet was cut along the axial direction, and the cut surface was polished and etched to reveal the sump profile. The temperature distribution was measured in the vertical direction at different radii from the billet center axis according to the following procedure. The tip of a K-type thermocouple was attached to the one end of a thin metal wire, while the other end of the wire was fixed at the bottom block of DC caster before starting the casting. The wire was stretched vertically in such a way that the thermocouple tip was positioned initially above the hot-top unit. Hence, as the billet moves down during the casting, the thermocouple tip passed through the molten metal in the hot-top, sump, and mushy zone that made it possible to measure the temperature distribution in the vertical direction of billet.

## 3. Mathematical Model

The main aim of the present model was to predict flow pattern and temperature distribution in the sump of DC caster. In the case of conventional DC casting, the model includes the balance equations of mass, momentum, and heat transfer for fully liquid and mushy regions, and heat transfer for solid phase region. In the case of ultrasonic DC casting, a model describing cavitation-driven acoustic streaming in liquid was added to the system of balance equations.

### 3.1. Acoustic Streaming

Acoustic cavitation and acoustic streaming are interrelated phenomena, and therefore, they should be modeled within a single conceptual framework. In the present study, we used the model that was reported in our earlier study [12]. Therefore, only a brief explanation will be given here. The main simplifications and assumptions used for the simulations are as follows:

(1) All calculations were performed at a constant size of cavitation bubble, which can be interpreted as a size averaged over the ultrasound wave cycle.
(2) Cavitation bubbles were assumed to be filled with air or hydrogen when ultrasound was irradiated in water or molten aluminum, respectively. Physical properties of gases and liquids were set to be temperature independent.
(3) As the bubble volume ratio is very low, the two-phase liquid-bubble flow was treated as a single-phase flow.
(4) The fluids were assumed to be incompressible and Newtonian
(5) Liquid flow, cavitation zone, and heat transfer were assumed to be axially symmetric.
(6) Thus, the present model is incapable of simulating the cavitation phenomena themselves, but it can predict and model several important phenomena related to the acoustic cavitation. These phenomena include the sound pressure distribution, cavitation zone formation and acoustic streaming generation.

Propagation of ultrasound wave in a bubbly liquid can be described by the linearized Helmholtz equation as follows [15]:

(1) $$\nabla^{2}P+k_{m}^{2}P=0$$

where *P* is the sound pressure amplitude and *k*_m_ is the complex wave number defined as:

(2) $$k_{m}^{2}=\frac{\omega^{2}}{c^{2}}\left(1+\frac{4\pi c^{2}NR_{0}}{\omega_{0}^{2}-\omega^{2}+2ib\omega }\right)$$

where *i* is an imaginary unit. This equation reveals that the wave number is dependent on the wave angular frequency, ω, resonant frequency of bubble, ω_0_, sound velocity in liquid, *c*, radius of undisturbed bubble, *R_0_*, damping factor, *b*, and bubble number density, *N*. The bubble resonant frequency *ω*_0_ can be determined from Equation (3):

(3) $$\omega_{0}^{2}=\frac{p_{0}}{\rho R_{0}^{2}}\left(Re\Phi -\frac{2\sigma }{R_{0}p_{0}}\right)$$

where *ρ* is the liquid density, *p*_0_ is the ambient pressure, σ is the surface tension, *Re* is the real part of *Φ* and *Φ* is the complex dimensionless parameter, which is the following function of the specific heat ratio of gas, γ, and a dimensionless parameter, χ

(4) $$\Phi =\frac{3\gamma }{1-3\left(\gamma -1\right)i\chi \left[{\left(i/\chi \right)}^{1/2}coth{\left(i/\chi \right)}^{1/2}-1\right]}$$

The latter is given as:

(5) $$\chi =D/\omega R_{0}^{2}$$

where *D* is the thermal diffusivity of gas. The damping factor *b* can be defined as:

(6) $$b=2\mu /\rho R_{0}^{2}+p_{0}Im\Phi /2\rho \omega R_{0}^{2}+\omega^{2}R_{0}/2c$$

where *μ* is the fluid dynamic viscosity. 

The bubble number density, *N* is the following function of bubble radius and volume fraction of bubbles, β.

(7) $$N=\frac{3\beta }{4R_{0}^{3}\pi }$$

The volume fraction was considered to be proportional to the absolute value of the sound pressure magnitude, |P|.

(8) β=C|P|

where the constant C was set to 2 × 10^−9^. Both the relation (8) and the value of constant C were suggested by Jamshidi [15]. A constant value of *R_0_* = 200 μm was used in the present study.

Thus, in the present model, number density and volume fraction of cavitation bubbles are fully governed by the amplitude of sound pressure, frequency of ultrasound waves as well as bulk and surface properties of fluid. Obviously, both the number density and volume fraction of cavitation bubbles reach their maximum values in the vicinity of sonotrode tip where the sound pressure amplitude is the highest. Ultrasound waves are incident on the surface of cavitation bubbles forcing them to move away from the tip along the ultrasonic wave propagation. These moving bubbles entrain the surrounding liquid into a stream motion, which is commonly termed acoustic streaming. It is noteworthy that this hypothesis has often been used to explain the origin of acoustic streaming [21,22]. In a real situation, when cavitation bubbles are oscillated, the interaction between these oscillating bubbles and ultrasound waves is rather complicated, and force acting on the bubbles in this case is called the primary Bjerknes force. However, as in the present model the size of bubbles is kept constant, expression for the force may be significantly simplified. If the bubble is small, so that the change of the spatial gradient of sound pressure ∇*P* within the bubble volume is negligible, the force, *F* acting on the bubble can be expressed in terms of ∇*P* and the bubble volume, *V* [23]:

(9) F=−〈V∇P〉

where the bracket indicates the time averaging. As has been discussed in our earlier work [12], in the case of enough strong acoustic field under the sonotrode tip, this equation can be further simplified and rearranged to give the final form usable in the present simulation:

(10) F=−β∇|P|

where β is the above-mentioned bubble volume fraction.

This equation was incorporated into the Navier-Stokes equation as an external volumetric force to simulate the acoustic streaming. The absolute value of sound pressure magnitude, |P|was calculated using the above set of Equations (1)–(8). The following boundary conditions were used.

(1) Liquid flow velocity: no-slip and slip conditions for liquid flow at the solid walls and free surface, respectively.
(2) Sound pressure: full reflection condition at the solid surfaces.

### 3.2. Solidification Model

As has been mentioned above, the mathematical model, proposed in the present study, attempts to predict unsteady flow and heat transfer phenomena during the DC casting process. In order to overcome the computation time inconvenience, a number of simplifications were made. Particularly, temperature and composition dependences of the physical properties of melt and solidified phases were ignored. Instead, average values for Al-17%Si alloy were applied in the present model. Moreover, the mushy zone was assumed to have the same porous morphology, without subdividing it into slurry and solid network parts. 

In the present study, the solidification model uses an enthalpy-porosity method of error-function model without considering shrinkage phenomena [24]. Additionally, the unsteady phenomena, including the movement of bottom block and solidified zone, are taken into account. The following porosity drag force is introduced into the Navier-Stokes equation as an external force:

(11) $$F=-C^{\prime }\frac{{\left(1-\alpha \right)}^{2}}{\alpha^{3}+b}\left(u-u_{b}\right)$$

where *C*′ is the drag constant, *α* is the solid fraction, *b* is the numerical stabilizing constant, ***u*** is the velocity, and ***u***_b_ is the bottom block velocity. In the present model, the moving coordinate was used only for the solidified region. The solid fraction *α* is described as:

(12) $$\alpha =\frac{1}{2}\mathrm{erf}\left(\frac{4\left(T-T_{m}\right)}{T_{l}-T_{s}}\right)+\frac{1}{2}$$

where erf indicates the error function, *T* is the temperature, *T*_m_ is the mean temperature between the solidus, *T_s_,* and liquidus, *T_l_,* temperatures. 

The following latent heat term is also introduced in the energy equation as:

(13) $$S=-\rho L\frac{4\exp\left(-{\left(\frac{4\left(T-T_{m}\right)}{T_{l}-T_{s}}\right)}^{2}\right)}{\left(T_{l}-T_{s}\right)\sqrt{\pi }}\cdot \left(\frac{\partial T}{\partial t}+u\cdot \nabla T\right)$$

where *S* is the latent source term due to phase change and *L* is the latent heat. 

Physical properties of molten metal necessary for the simulation are presented in Table 1. It is to be noted that no turbulent model was included in the present simulation. Instead, direct numerical simulation was applied over the fluid computational domain with very fine mesh. The following heat transfer coefficients were set as boundary conditions at the mold inner surface (primary cooling zone, PCZ) and water-cooled part of biller surface (secondary cooling zone, SCZ). Primary cooling zone, *h_m_* = 10 kW/m^2^⋅K, secondary cooling zone, *h_b_* = 10 kW/m^2^⋅K. The zone locations are indicated in Figure 3 as PCZ and SCZ, respectively.

## 4. Results 

### 4.1. Verification of Acoustic Streaming Model 

**Figure 4:**
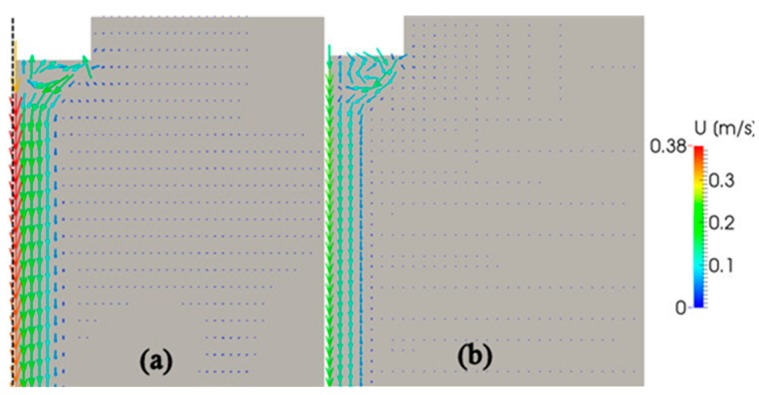
Predicted velocities of acoustic streaming in water (**a**) and molten aluminum (**b**).

Figure 4 presents typical numerical results on acoustic streaming velocity in water (Figure 4a) and molten aluminum (Figure 4b). The vibration amplitude of sonotrode tip was set to 50 μm (p-p), which is the same value as in the above-mentioned PIV experiment. Predicted velocity vectors for water indicate a reasonable agreement with the experimental observations (Figure 2), although the acoustic flow obtained by the PIV measurement is slightly diffusive compared with the predicted one. Pattern of acoustic streaming in molten aluminum is very similar to that in water; however, the streaming velocity is significantly slower. The reason for this has been discussed in our earlier paper [12].

### 4.2. Temperature Distribution and Sump Characteristics

**Figure 5:**
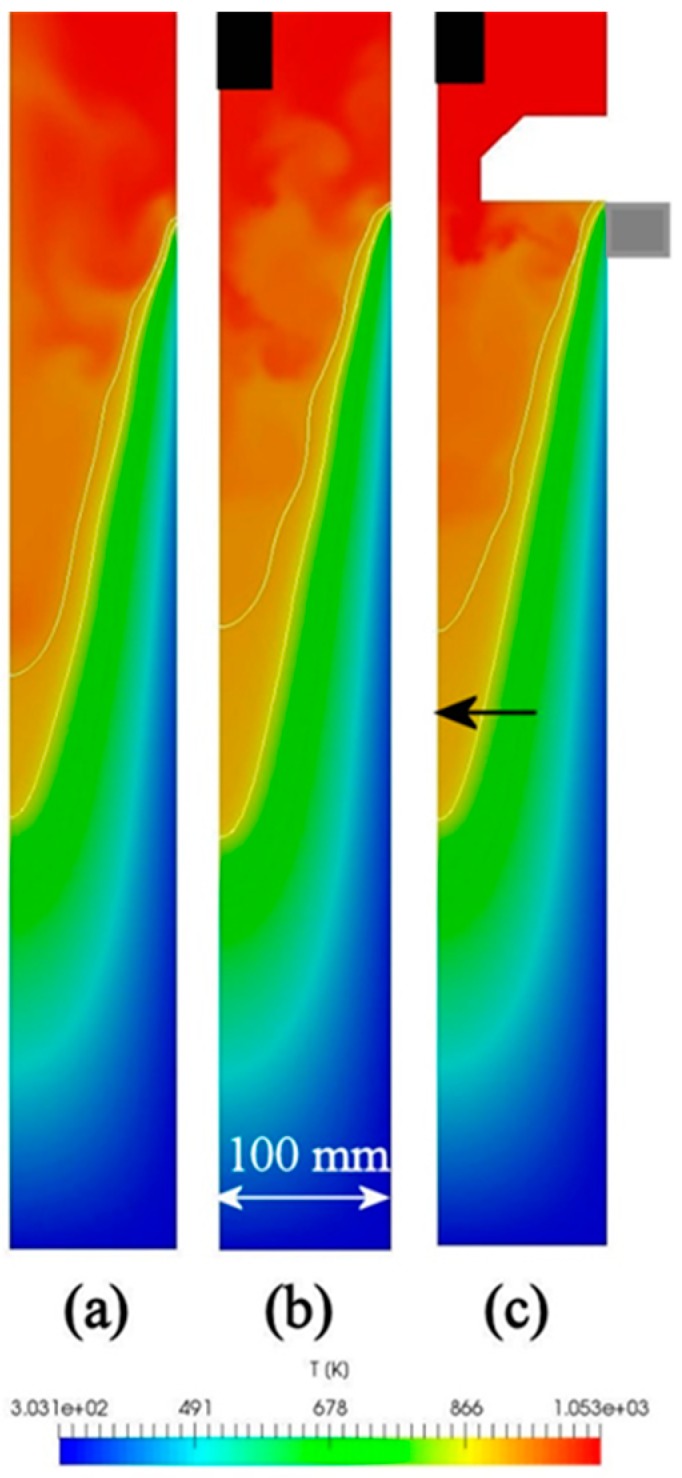
Distribution of temperature in sump. (**a**) Conventional casting, (**b**) UST HT casting, (**c**) UST FRHT casting.

**Figure 6:**
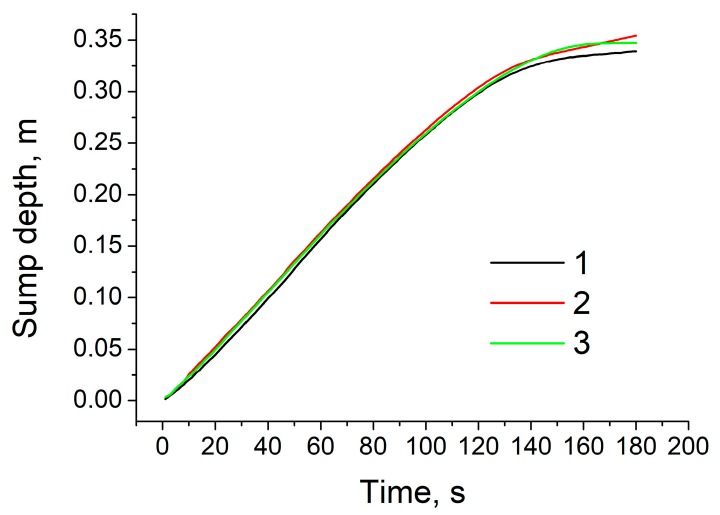
Time variation of sump depth. 1: conventional casting; 2: UST HT castingmaterials-625822 3: UST FRHT casting.

**Figure 7:**
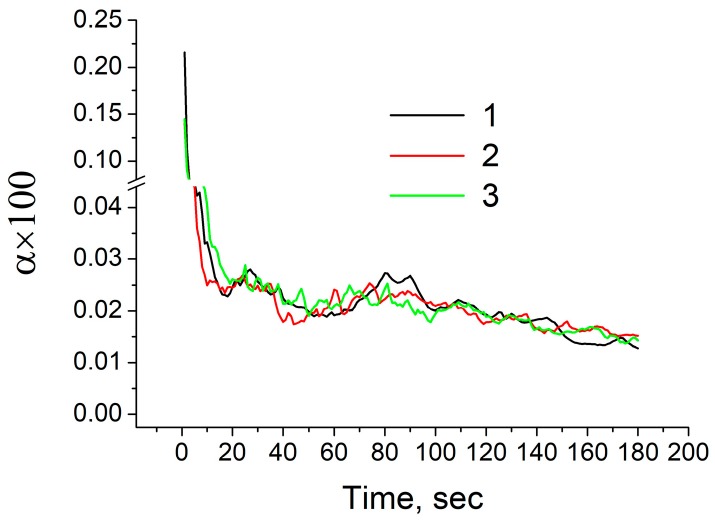
Time variation of dimensionless volume of mushy zone. 1: conventional casting; 2: UST HT casting; 3: UST FRHT casting.

Next, using the above mathematical models, unsteady numerical simulations were conducted to predict evolution of the sump depth and profile over time as well as time-dependent distributions of temperature and velocity in the sump during the casting process. The total time of simulation was set to 3 min with an interval of 1 s. Figure 5 shows typical temperature contours in the sump in the last 180th second of simulation for the above-mentioned three cases. These contours are snapshots of supplemental Videos S1–S3 showing the time evolution of sump and temperature. The diameter of billets was 203 mm and the height of the mold was 30 mm, as shown by a grey rectangle in Figure 5c. The melt initial temperature and casting speed was set to 780 °C and 200 mm/min, respectively. Two black rectangles at the upper part of Figure 5 indicate the location of sonotrode tip. Two white curves at each temperature contour indicate the locations of the liquidus and solidus profiles. Therefore, the area between these two lines corresponds to the mushy zone where the solid and liquid phases coexist. The results reveal that the sump depth and profile are independent of casting method. In the case of UST in the mold (Figure 5b), the sump looks slightly deeper as compared to the other conditions. However, this difference is rather small, as indicated in Figure 6. This figure shows time variation of the sump depth for all three conditions. The sump depth was defined as the distance from the bottom edge of the mold to the deepest location at the liquidus profile. The data of Figure 6 suggests that the sump depth increases rapidly in the beginning of casting, but then the increase gradually slows down and the depth becomes almost constant after 3 min of casting. Moreover, in Figure 5, the mushy zone formed during the conventional casting (Figure 5a) looks smaller than those formed under application of ultrasound oscillations. However, time variations of dimensionless volume of mushy zone, α presented in Figure 7 clearly reveal that this volume varies with time approximately in the same way for all casting methods examined in this study. The values of α were non-dimensionalized relative to volume of melt flowing into the sump from the mold. The only difference is that fluctuations of α values occurs more frequently under the UST conditions compared to the case of conventional casting. The reason for that will be discussed below.

**Figure 8:**
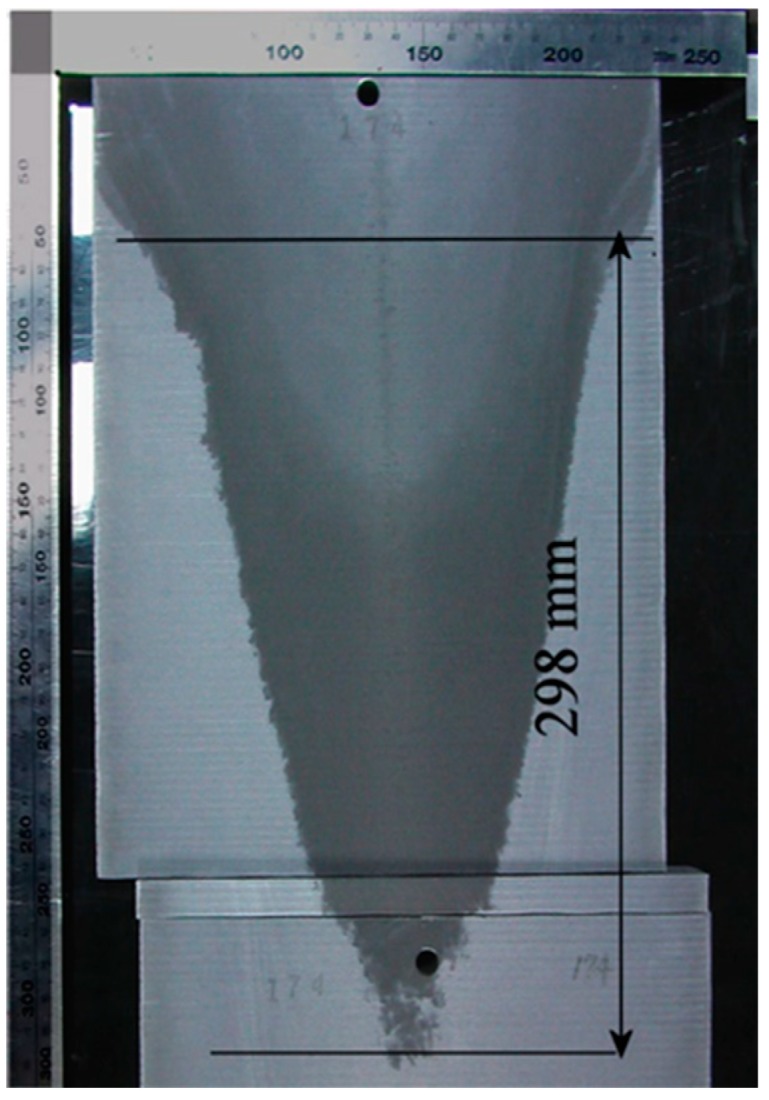
Profile of the sump in a billet produced by UST FRHT casting.

Figure 8 presents the sump depth and profile measured experimentally in the case of UST FRHT casting. The casting conditions were the same as mentioned above. As it was difficult to identify the location of the mold from this picture, the sump depth can be measured only roughly. Under the given conditions, the sump depth is 300 mm approximately. It is to be noted that this value is much greater than those reported in the literature for close sizes of billet and the similar casting speed [25]. This is because in our experiments, a different alloy and much higher temperature of casting was used. However, as can be seen from Figure 8, at the lower part of the sump the mushy zone loses its continuity, suggesting that this is a partly or fully coherent solid part of the mushy zone. Therefore, the depth of sump may be shorter than 300 mm. The black horizontal arrow in Figure 5c shows the point corresponding to the 300 mm depth. It is clearly seen that this point is located inside the predicted mushy zone. This finding suggests a good agreement and predictability for our model. As the mushy zone was interpreted as being a porous solid phase, the predictions of sump depth give underestimated values in the present model. In the actual casting process, as a part of mushy zone is in form of flowable slurry, the actual sump should be deeper.

### 4.3. Distribution of Velocity in the Sump

**Figure 9:**
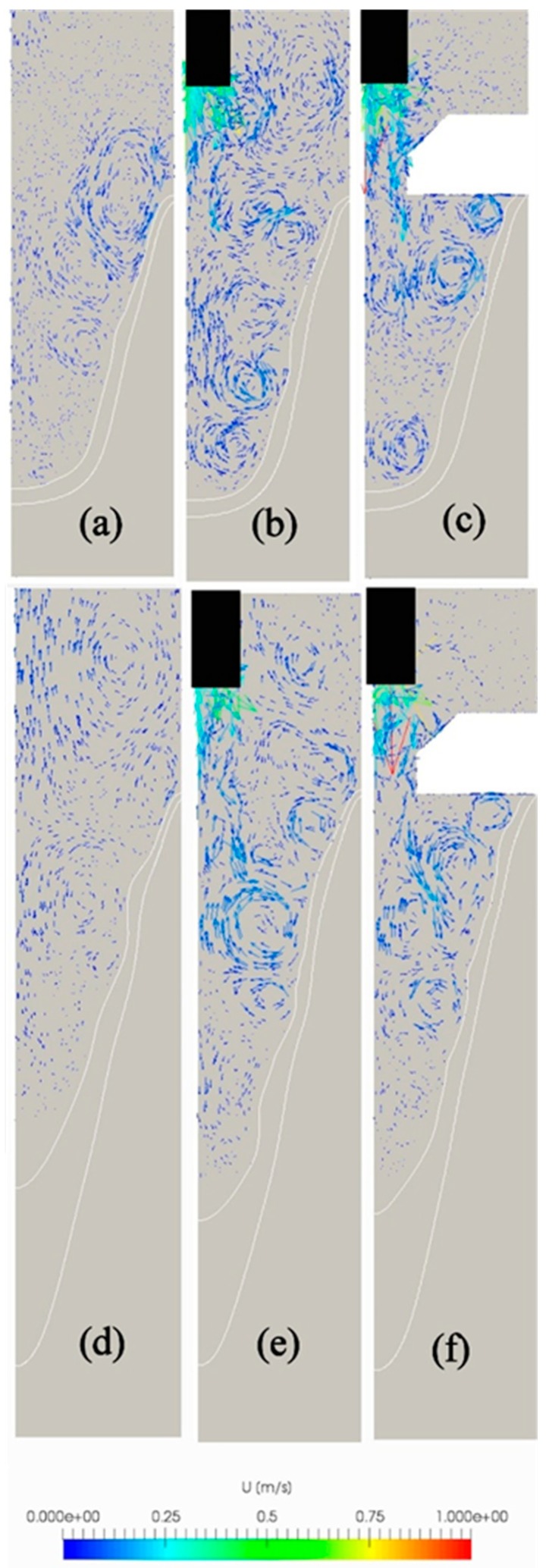
Predicted velocities in the sump during its evolution. (**a**,**d**) Conventional casting, (**b**,**e**) UST HT casting, (**c**,**f**) UST FRHT casting.

The results of numerical simulation revealed that the velocity characteristics are considerably changed with the application of ultrasound vibrations. Figure 9 presents two typical sets of velocity vectors over the sump computed under conditions of conventional casting (a,d), UST HT casting (b,e), and UST FRHT casting (c,f) in the 65th (a,c) and 142nd (d,f) seconds of casting. These images are snapshots of supplemental Videos S4–S6, where more details on the velocity vectors can be found. In the case of conventional casting, the magnitude of velocity vectors is considerably smaller than in the cases of ultrasonic assisted casting. However, what is more important is that the ultrasonic vibrations make the melt flow inside the sump more turbulent. This can be readily seen from Figure 9b,c,d,f; a lot of eddies of small sizes are formed in the sump bulk and near the solidification front including the area close to the mold surface. The difference in the flow pattern between the conventional and UST castings is especially significant in the upper half of the sump. The eddy formation results in thinning of the mushy zone layer, as can also be seen from this figure. This phenomenon can cause the cooling rate to become higher that can contribute to the refinement of microstructure. The simulation results suggest that the eddy formation can occur also during the conventional casting as shown in Figure 9a. However, the eddy size in this case is much larger as compared to the UST casting cases. Another important finding is that the eddy formation becomes weaker with the distance from the sonotrode. As can be seen from Figure 9e,f, no eddies are produced in the lower part of the sump.

## 5. Discussion

The results of numerical simulations revealed that introduction of ultrasonic vibrations into the melt, flowing through the hot-top unit, has little or no effect on the temperature distribution and sump profile, but has a considerable effect on the melt flow pattern in the sump. Below is a discussion regarding the obtained simulation results and an additional verification of them versus experimental observations. 

The reason why there is almost no difference in the temperature distribution, volume of mushy zone, and profile of the sump between these three casting methods is assumed to be as follows. In the present simulation, all boundaries except for the primary cooling surface (mold inner surface) and secondary cooling surface (water cooled part of billet) were set to be adiabatic. However, in the actual DC casting process, a part of the heat can escape through the melt free surface and walls at the hot-top unit. Moreover, it is well known that irradiation of ultrasound in liquids including molten metals is accompanied by generation of heat, which can be added to the melt [1]. In the present study, the heat generation was ignored. The fact that heat was lost through the melt free surface and hot-top unit walls can be demonstrated by the following experimental observations.

**Figure 10:**
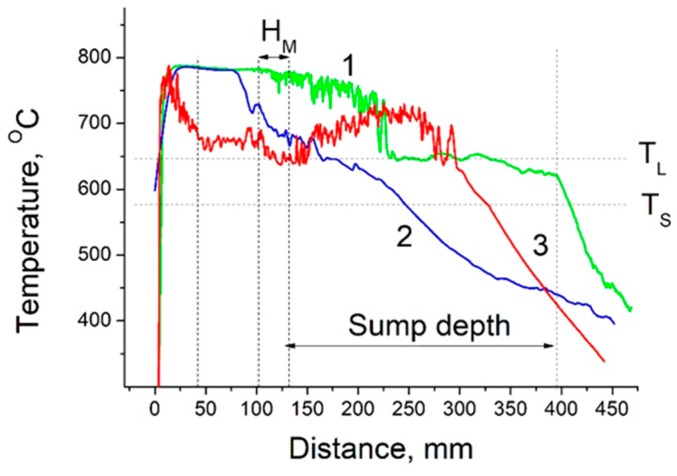
Variation of the melt temperature with distance from the melt surface. 1,2—FRHT without ultrasound irradiation, 3—FRHT with ultrasound irradiation.

Figure 10 shows variations of temperature along the vertical distance when the thermocouple tip passes through the molten bath above the partition plate, partition plate (Figure 3), melt in sump, mushy zone and finally enters the body of solidified billet. In this figure, vertical broken line in the left part indicates location of the top surface of partition plate, two vertical broken lines in the middle part correspond to location of mold of height H_M_ = 30 mm, and vertical broken line in the right part shows an approximate location of the sump depth. Two horizontal broken lines indicate the liquidus, T_L_, and solidus, T_S_, temperatures. The measurement details and hot-top arrangement have been described in the above section. The measurements were conducted using the flow-restricting hot-top without and with ultrasound application. In the first case, two thermocouples were set in such a way that the first one passed along the billet centerline, while the second one passed at a distance of half-radius (R/2) from the center line. In the case of UST casting, the temperature measurements were performed only at the half-radius distance because the presence of sonotrode made is impossible to conduct the measurements along the centerline. In the casting without UST, the thermocouples enter the melt at a temperature of 780 °C, which remains almost the same till the thermocouple tips enter the melt in the sump (centerline location) or the partition plate (half-radius location). In the latter case, the measurements show that temperature inside the partition plate is reduced by 70 °C. After entering the melt inside the sump, the thermocouple indications become significantly dependent on the locations. At the center line (line 1), the melt temperature is slowly decreased until the upper boundary of the mushy zone is reached. It should be pointed that in this area, the temperature shows fluctuations that is in accordance with the results of numerical simulations. After entering the mushy zone, the temperature is abruptly decreased down, and then remains constant and close to T_L_. This part of mushy zone is assumed to be fully flowable and its length is estimated to be about 100 mm. Then, as the thermocouple tip enters deeper into the mushy zone, temperature starts to lower (this point is shown by arrow in Figure 10), and finally drops to T_S_. Most likely, these parts correspond to slightly coherent semisolid and fully coherent solid areas within the mushy zone. If one defines the sump as a zone in which molten metal can still flow, the sump depth becomes as shown by the right vertical line in Figure 10. At the R/2 location (line 2), the temperature drops more rapidly, and therefore, reaches the solidus temperature at a much shorter distance from the melt free surface compared to that at the centerline. However, under application of ultrasonic vibrations, the variation of temperature with distance is drastically changed (line 3). Firstly, the temperature fluctuates to a much greater extent than that during the casting without UST. This finding is in good agreement with the numerical predictions suggesting the generation of small eddies, especially at areas close to the mushy zone. Secondly, the temperature of the melt above the partition plate is significantly decreased with the distance from the melt surface. The most likely reason of this temperature drop is acoustic streaming which occurs in the melt above the partition plate enhancing heat loss through the melt free surface and hot-top walls. As a result, the temperature inside the partition plate becomes much lower as compared to that without UST. Thirdly, the temperature in the sump near the mold surface is decreased down to the liquidus temperature T_L_, but then significantly increased up to 720 °C and decreased again to T_L_ with the distance from the partition plate. Finally, the temperature abruptly drops from T_L_ to T_S_ within a distance which is much shorter than in the case without UST suggesting a significant thinning of the mushy zone at the R/2 location. Additionally, these findings suggest that the sump, formed under conditions of ultrasound application, should have substantially greater width and, probably, depth as compared to the case without UST. However, these findings do not support the results of numerical simulation on the sump profile and temperature variation. Thus, although more experimental observations are needed to clarify this discrepancy, the experimental measurements of the present study suggest that our acoustic streaming model needs further improvements, particularly in terms of more accurate descriptions of boundary conditions and mushy zone characteristics.

It should be pointed out that the method of ultrasonic treatment of the melt in the flow-restricting hot-top unit has been developed in attempt to improve the efficiency of cavitation treatment of molten metal, particularly refiner particles in the melt, and to control the melt flow pattern in the sump. As reported in our earlier work [10], this method allows us to get two effects at the same time, namely the structural refinement and uniformity. In light of the results of the present numerical simulation, acoustic streaming, when occurs in the sump of molten aluminum, results less in the enhancement of convective flow than in the generation of turbulent eddies of relatively small sizes in the sump. It should be noted that this effect differs from that reported by Lebon et al. [19]. In their study, the tip of a sonotrode was positioned closer to the sump bottom, and therefore, the role of convection flow driven by the acoustic streaming was much greater than under conditions of our study. It is assumed that the generation of turbulent eddies in the sump is favorable from two points of view. First, the eddies can improve the dispersion of refiner particles and enhance their transport to the mushy zone. Second, as can be seen from Figure 9, small eddies are produced in the immediate vicinity of the mold surface, especially in the case of UST in the flow-restricting hot-top. This may be effective in controlling the billet surface defects, particularly the ripple mark. Our preliminary observations showed that the appearance of ripple marks on the billet surface are remarkable changed depending on the casting method and conditions. These issues are the subject of our ongoing research.

## 6. Conclusions

In the present work, melt flow pattern and temperature distribution in the sump of aluminum billets produced in a hot-top equipped DC caster conventionally and with ultrasonic irradiation were investigated, with the main emphasis being placed on the effects of acoustic streaming and hot-top unit type. As direct observations of flow phenomena in molten aluminum is impossible, the acoustic streaming characteristics were predicted by using the earlier developed numerical model verified against results of water model experiments. Then, the acoustic streaming model was applied to develop a simplified numerical model, which was capable, for the first time, of simulating unsteady phenomena in the sump during DC casting process. The results of numerical simulations were verified against measurements performed on an ultrasonic-assisted DC caster producing 203 mm billets of Al-17Si alloy at a casting speed of 200 mm/min. The results of the present study reveal the following.

(1) According to the numerical simulations, the evolution of the melt sump continues about 3 min irrespective of the ultrasound application and hot-top type. The final depth and the shape of the sump are also independent of the casting conditions examined.
(2) Irradiation of ultrasound into the melt at the hot-top unit results in more frequent fluctuations of the temperature and mushy zone volume around their mean values as compared to the conventional DC casting case, although the mean values themselves remain the same in both the cases.
(3) The pattern of melt flow in the sump is drastically changed with the ultrasound application. The flow velocity becomes faster in general and a lot of relatively small eddies are produced in the sump bulk and near the mushy zone. The latter causes frequently repeated thinning of the mushy zone layer.
(4) The experimental verification revealed approximately the same sump depth and shape as those predicted by the numerical simulations, and confirmed the frequent and large fluctuations of the melt temperature during ultrasound irradiation. However, the measurements of the temperature distribution in the sump showed a significant difference between the cases of castings without and with ultrasound irradiation. This suggests that the present mathematical model should be further improved, particularly in terms of more accurate descriptions of boundary conditions and mushy zone characteristics.

## Figures and Tables

**Table 1:** Model parameters used for the DC casting simulation.

Parameter	Unit	Quantity
Dynamic viscosity	kg⋅m^−1^⋅s^−1^	5.472 × 10^−7^
Density	kg⋅m^−3^	2330
Coefficient of thermal expansion	-	2.1 × 10^−5^
Heat capacity	kJ⋅kg^−1^⋅K^−1^	1.282
Thermal conductivity	W⋅kg^−1^⋅K^−1^	80
Heat of fusion	kJ⋅kg^−1^	450
Prandtl number	-	0.0166
Blake threshold	Pa	1.0 × 10^5^
Liquidus temperature	K	920.15
Solidus temperature	K	850.15
Ultrasound frequency	kHz	20
Sound speed	m⋅s^-1^	4650

## Supplementary Materials

The following are available online at https://www.mdpi.com/1996-1944/12/21/3532/s1, Video S1: time variation of sump profile and temperature for conventional casting; Video S2: time variation of sump profile and temperature for UST HT casting; Video S3: time variation of sump profile and temperature for UST FRHT casting; Video S4: time variation of sump profile and melt flow for conventional casting; Video S5: time variation of sump profile and melt flow for UST HT casting; Video S6: time variation of sump profile and melt flow for UST FRHT casting.
