# Supplementary figures and images for: Role of Acoustic Streaming in Formation of Unsteady Flow in Billet Sump during Ultrasonic DC Casting of Aluminum Alloys
Source: Materials (Basel). 2019 Oct 28;12(21):3532. doi: 10.3390/ma12213532 (PMC6862103; doi:10.3390/ma12213532)

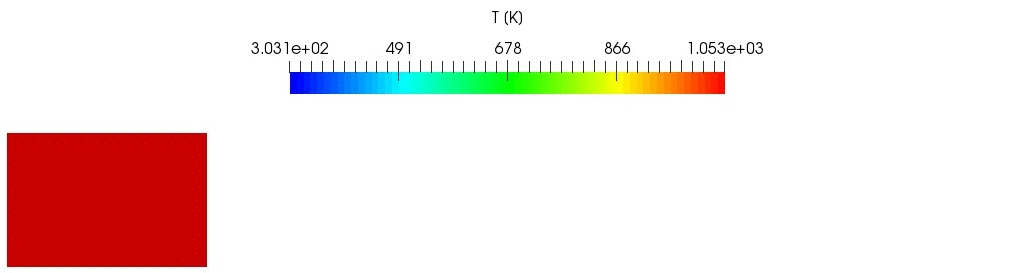

Supplement: Supplementary file 1 [file materials-12-03532-s001.zip › Sergey Komarov-supplementary materials/S1.gif]

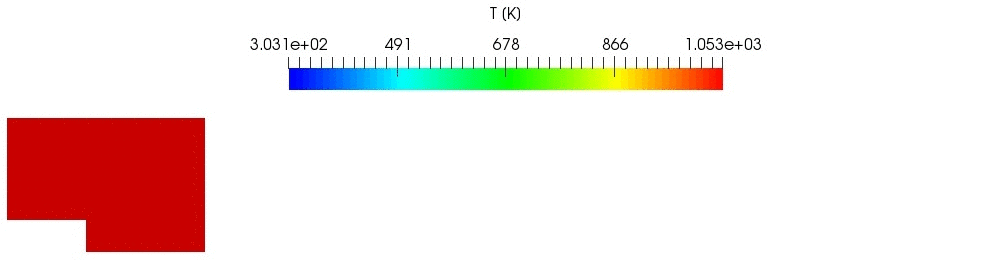

Supplement: Supplementary file 1 [file materials-12-03532-s001.zip › Sergey Komarov-supplementary materials/S2.gif]

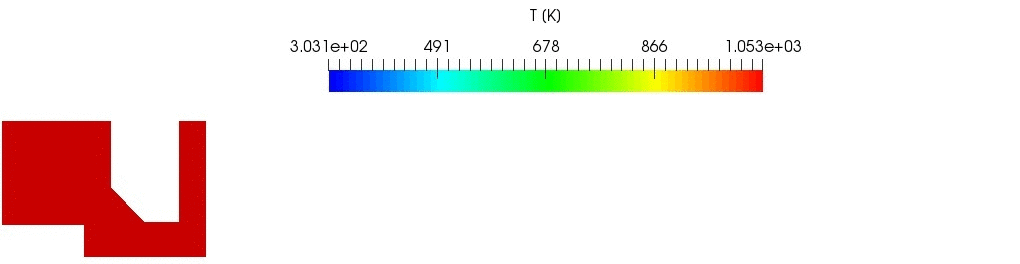

Supplement: Supplementary file 1 [file materials-12-03532-s001.zip › Sergey Komarov-supplementary materials/S3.gif]

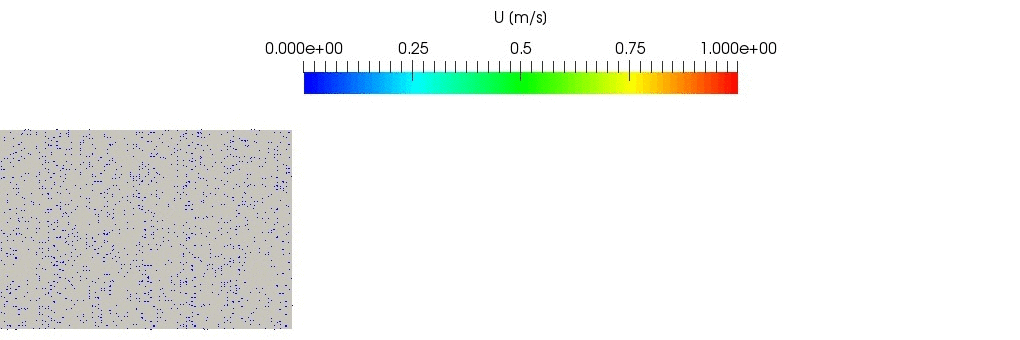

Supplement: Supplementary file 1 [file materials-12-03532-s001.zip › Sergey Komarov-supplementary materials/S4.gif]

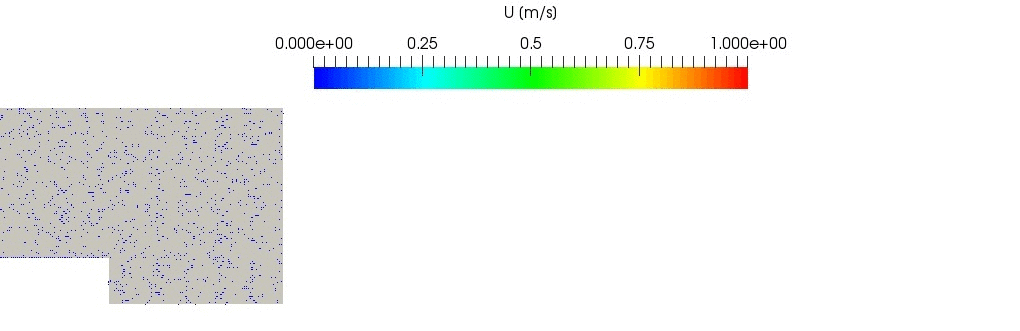

Supplement: Supplementary file 1 [file materials-12-03532-s001.zip › Sergey Komarov-supplementary materials/S5.gif]

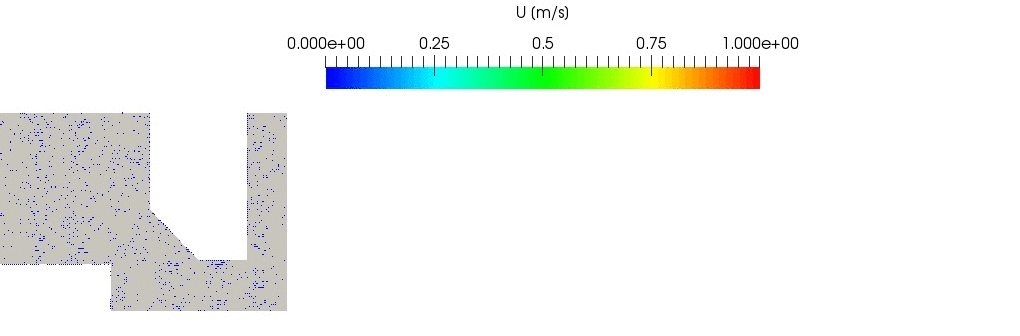

Supplement: Supplementary file 1 [file materials-12-03532-s001.zip › Sergey Komarov-supplementary materials/S6.gif]
